# Dietary supplementation with calcium propionate could beneficially alter rectal microbial composition of early lactation dairy cows

**DOI:** 10.3389/fvets.2022.940216

**Published:** 2022-07-26

**Authors:** Fan Zhang, Yiguang Zhao, Yue Wang, Hui Wang, Xuemei Nan, Yuming Guo, Benhai Xiong

**Affiliations:** ^1^State Key Laboratory of Animal Nutrition, Institute of Animal Sciences, Chinese Academy of Agricultural Sciences, Beijing, China; ^2^State Key Laboratory of Animal Nutrition, College of Animal Science and Technology, China Agricultural University, Beijing, China

**Keywords:** calcium propionate, dairy cows, early lactation, immune function, fecal microbial composition

## Abstract

Dietary supplementation with calcium propionate can effectively alleviate negative energy balance and hypocalcemia of dairy cows in early lactation. The objective of this study was to investigate the effects of calcium propionate feeding levels on the immune function, liver function, and fecal microbial composition of dairy cows in early lactation. Thirty-two multiparous Holstein cows were randomly assigned to four treatments after calving. Treatments were a basal diet plus 0, 200, 350, and 500 g calcium propionate per cow per day throughout a 5-week trial period. Cows were milked three times a day, and blood was sampled to measure immune function and liver function on d 7, 21, and 35. The rectal contents were sampled and collected on d 35 to analyze the microbial composition using 16S rRNA gene sequencing. The results indicated that increasing amounts of calcium propionate did not affected the serum concentrations of total protein, IgG, IgM, and calcium, but the concentrations of albumin and IgA changed quadratically. With the increase of calcium propionate, the activity of serum alanine transaminase and aspartate aminotransferase increased linearly, in contrast, the activity of alkaline phosphatase decreased linearly. Moreover, dietary supplementation with increasing levels of calcium propionate tended to quadratically decrease the relative abundance of *Firmicutes* while quadratically increased the abundance of *Bacteroidetes*, and consequently linearly decreased the *Firmicutes*/*Bacteroidetes* ratio in the rectal microbiota. Additionally, the supplementation of calcium propionate increased the relative abundances of *Ruminococcaceae_UCG-005* and *Prevotellaceae_UCG-004* linearly, and *Ruminococcaceae_UCG-014* quadratically, but decreased the relative abundances of *Lachnospiraceae_NK3A20_group* and *Family_XIII_AD3011_group* quadratically. Compared with the CON group, the calcium propionate supplementation significantly decreased the relative abundance of *Acetitomaculum* but increased the abundances of *Rikenellaceae_RC9_gut_group* and *Alistipes*. In summary, these results suggested that the supplementation of calcium propionate to dairy cows in early lactation could beneficially alter the rectal microbiota.

## Introduction

In early lactation, dairy cows often experience the challenges of both metabolic and infectious diseases (1). Compared with the dry period, the initiation of lactation in dairy cows requires extra energy and nutrients from diet (2). However, the nutrient requirements for milk production and maintenance increase faster than the supply *via* feed intake in the early lactation. Although body tissue mobilization can partly compensate for the prevailing lack of energy and nutrients, dairy cows in this period still often suffer several production diseases, particularly ketosis (3), fatty liver (4), and milk fever (5). These metabolic diseases play an etiological role in decreasing feed intake and milk production, reducing fertility, impairing immune function, and increasing cow culling rate (2, 6). The negative nutrient balance also greatly increases the risk of other postpartum diseases including mastitis, metritis, retained placenta, displaced abomasum, and uterine prolapse, which may further increase the incidence of removal and economic losses (7, 8). Therefore, these metabolic disorders should be prevented or treated during the transition period. Propionate, one of the major fermentation products of rumen microorganisms from carbohydrates, is the main substrate for gluconeogenesis (9). Oral supplementation to dairy cows with large amounts of soluble calcium (Ca) is possible to force Ca pass across the intestinal tract by passive diffusion between intestinal epithelial cells and blood to prevent hypocalcemia. It has been found that the doses of Ca between 50 and 125 g/d can obtain the optimal production effects for dairy cows in early lactation (10). Synthetically, calcium propionate, which is less injurious to tissues and not acidogenic, could dissociate into Ca ions and propionate in the rumen (11, 12). Dietary supplementation with calcium propionate to dairy cows in early lactation can increase milk production (13, 14), decrease the incidences of subclinical hypocalcemia and milk fever (11, 15), and improve energy status (16).

During the early lactation, dairy cows also experience immune dysregulation, which is recognized as the most common cause of metritis and mastitis (17). Due to the increase in cell respiration rate, increased milk production of dairy cows often causes oxidative stress, which leads to some infectious and non-infectious diseases (18). In our previous study, we have found that dietary supplementation with 350 g/d calcium propionate improved milk performance and affected the regulation of serum bile acids metabolism (19). However, the effects of calcium propionate on the health throughout early lactation have not been elucidated. The gut microbiota, which can be affected by feed intake, host genetics, age, environment, and stress, plays an essential role in maintaining the health of gastrointestinal tract (20), improving nutrient utilization, and enhancing gut function. The imbalance in the intestinal microbiome can also cause inflammatory responses (21). However, as an effective additive for dairy cows to alleviate negative energy balance (NEB) and hypocalcemia, the effects of calcium propionate on the gut microbiota have not been studied. 16S rDNA sequencing is commonly used to analyze the microbial community composition information. Therefore, the objective of this study was to investigate the effects of calcium propionate feeding levels on the immune function, liver function, and fecal microbial composition of dairy cows in early lactation. These data would be helpful for evaluating the functions of calcium propionate in alleviating negative nutrient balance of dairy cows in early lactation.

## Materials and methods

All animals used in this study were approved by the Care Committee of the Chinese Academy of Agricultural Sciences (Protocol number: IAS2020-93; Beijing, China), and all the experimental procedures were carried out according to the guidelines of the academy for animal research.

### Animals, diets, and experimental design

This study was conducted between September and December 2020 at a China-Israel demonstration dairy farm (Beijing, China). Thirty-two multiparous Holstein dairy cows were randomly distributed into 4 treatments (8 cows per group) balanced with the corrected 305-d milk yield (41.61 ± 1.61 kg/d, mean ± SD) in the previous lactation, parity (3.29 ± 0.18), body weight (779 ± 12.5 kg) and anticipated calving date in a randomized block design. The dairy cows entered the study from the first day after calving to d 35 in milk. The dairy cows in the control (CON) group were only fed a basal total mixed ration (TMR) diet which was formulated to meet the requirements for energy, protein, minerals, and vitamins based on the National Research Council (22) for dairy cows in early lactation. The other three groups: low calcium propionate (LCaP), medium calcium propionate (MCaP), and high calcium propionate (HCaP) were fed the same basal TMR diet and orally drenched with 200, 350, and 500 g/d of calcium propionate per cow, respectively. The calcium propionate was orally drenched by a stainless-steel pill propellant gun (Boehringer-Ingelheim, Ingelheim, Germany) 3 times a day after milking. The supplementation levels of calcium propionate were designed according to the previous studies of Liu et al. (16) and Martins et al. (14). The ingredients and nutrient composition of the basal diet is shown in Table 1. The calcium propionate was purchased from Jiangsu Runpu Food Technology Co. LTD (Lianyungang, Jiangsu, China). All cows were fed *ad libitum* three times a day at 7:00, 14:30, and 18:00 h and milked three times a day at 6:00, 14:00, and 22:00 h, respectively. The feed offered was adjusted daily according to the feed intake of the previous day and ensured 5–10% refusals. All cows were housed individually and had free access to feed and water. In the process of the experiment, three cows (one in the MCaP group for metritis and two in the CON group for left displaced abomasum) were eliminated, and the corresponding data were discarded. The procedures for sample collection and chemical analysis were similar to our previous study performed at the same farm (19).

**Table 1:** The ingredients and nutrient composition of the basal diet.

**Items**	
Ingredients, g/kg of DM ^a^	
Concentrate ^b^	419
Stem-flaked corn	35.8
Sprouting corn bran	21.9
Cottonseed	22.8
Fat power	11.4
Pelleted beet pulp	13.1
Megalac ^c^	4.95
Wet brewer grains	37.3
Alfalfa	99.0
Oat hay	21.6
Corn silage	313
Nutrient composition, % of DM unless otherwise stated	
Net energy for lactation ^d^, MJ/kg DM	7.20
Crude protein	17.7
Neutral detergent fiber	28.0
Acid detergent fiber	15.9
Ether extract	4.2
Ash	9.07
Calcium	0.85
Phosphorus	0.42

^a^1DM, dry matter.

^b^The concentrate was manufactured by Beijing Sanyuan Seed Technology Co., Ltd. (Beijing, China). The concentrate provided (per kg DM basis) 239.1 g CP; 132.0 g NDF; 74.0 gg ADF; 14.1 g Ca; 5.80 g P; 12.0 g K; 5.80 g Mg; 9.90 g Na; 46.25 mg Cu; 80.30 mg Fe; 136.76 mg Zn; 20,530 IU vitamin A; 3,548.5 IU vitamin D; 115.89 IU vitamin E; 21.89 g NaHCO_3_.

^c^The megalac was produced by Volac Wilmar Feed Ingredients, Ltd. (Hertfordshire, United Kingdom).

^d^Net energy for lactation was calculated according to NRC (22).

### Sample collection and analysis

The samples of the TMR were collected every week to determine the nutrient composition. The samples were dried for 48 h at 55°C in a forced-air oven and ground passed through a 1-mm screen for subsequent nutrient composition analysis as described by Zhang et al. (19). The dry matter (DM), crude protein (CP), ether extract (EE), ash, acid detergent fiber (ADF), calcium (Ca), and phosphorous (P) contents of the diet samples were determined according to the method of AOAC (23). The method of Van Soest et al. (24) was used to determine the content of neutral detergent fiber (NDF) with heat-stable α-amylase and sodium sulfite.

Blood samples of each cow were harvested on d 7, 21, and 35 from the coccygeal vein into 10-mL gel vacuum tubes before the morning feeding. Serum was obtained from the supernatant after the blood was centrifuged at 3,000 × g at 4°C for 15 min. The serum samples were then frozen at−20°C for later analysis. The serum concentrations of alanine transaminase (ALT), aspartate aminotransferase (AST), and alkaline phosphatase (ALP) were determined using commercial assay kits (Nanjing Jian Cheng Bioengineering Institute, Nanjing, China), according to the manufacturer's instructions (25). The concentration of serum total Ca was determined by using the colorimetric commercial kits (Shanghai Kehua Bio-Engineering CO., LTD, Shanghai, China). The concentrations of serum total protein (TP) and albumin (ALB) were measured by an automated biochemical analyzer (Hitachi 7080; Hitachi Valve Ltd., Tokyo, Japan). The concentrations of serum IgA, IgG, and IgM were determined by using bovine ELISA kits (Bethyl Laboratories, Montgomery, TX, USA). On d 35, six cows from each group were randomly selected to collect fresh fecal samples from the rectums. All fecal samples were immediately frozen in liquid nitrogen and then stored at−80°C for later microbial DNA extraction.

### Fecal microorganism DNA extraction, PCR amplification, and sequencing

The total DNA was extracted using an Omega Stool DNA kit (Omega BioTek, Norcross, GA, USA) according to the manufacturer's instructions. The purity and quality of the extracted DNA were checked using 1% agarose gels and a NanoDrop ND-1000 spectrophotometer (Thermo Fisher Scientific, Waltham, MA, USA). The PCR was used to amplify the V3-V4 hypervariable region of the bacterial 16S rRNA gene with the universal primers 338F (5'-ACTCCTACGGGAGGCAGCAG-3') and 806R (5'-GGACTACNNGGGTATCTAAT-3') (26). The PCR was performed in triplicate 25 uL reactions containing 2 uL DNA template, 12.5 μl 2 × Taq PCR MasterMix (Solarbio, Beijing. China), 1 μL Forward Primer (5 uM), 1 μL Reverse Primer (5 uM), 3 μL bovine serum albumin (2ng/μL), and 5.5 μL ddH_2_O. The amplification was started with a denaturation at 95 °C for 5 min, followed by 28 cycles at 95°C for 45 s, 55°C for 50 s and 72°C for 45 s, and a final extension at 72°C for 10 min. Triplicate PCR products of the same samples were mixed in equimolar ratios into a single tube. The PCR products were purified using an Agencourt AMPure XP Kit (Beckman Coulter, Brea, CA, USA). High-throughput sequencing was conducted using Illumina Analysis Pipeline Version 2.6 (Illumina, San Diego, CA, USA) finished by Allwegene Company (Beijing, China). The raw sequencing data of this study was deposited in the NCBI BioProject database under the accession number of PRJNA822933.

### Sequence processing and analysis

The raw sequencing data were filtered if the sequences were shorter than 120 bp, had a low-quality score ( ≤20), contained ambiguous bases, or did not exactly match the primer sequences and barcode tags. The assembled sequences were trimmed of primers and clustered into operational taxonomic units (OTUs) at a similarity level of 97% using UPARSE v7.1 (27). The phylogenetic affiliation of each 16S rRNA gene sequence was analyzed with Ribosomal Database Project (RDP) classifier against the SILVA128 database (28) using the confidence threshold of 80%. Alpha diversity indices (observed species, Chao1, PD_whole_tree, Shannon index, and Simpson index) of the ruminal bacterial communities were assessed with QIIME 1.8 based on the OTU information. The OTU rarefaction curve was also plotted in QIIME. To compare the differences of fecal bacterial communities among the four treatments, principal coordinate analysis (PCoA) plots for β-diversity were generated using the permutational analysis of variance (PERMANOVA) by the adonis function in R software vegan package based on Bray Curtis dissimilarity. R (v3.6.0) software was used for bar-plot diagram analysis based on the results of taxonomic annotation and relative abundance.

### Statistical analysis

All data were analyzed using the MIXED procedure of SAS (version 9.4, SAS Institute Inc., Cary, NC, USA) as a randomized complete block design with repeated measures. The statistical power for the samples size was > 0.8 with the significance level of 0.05 by the G^*^Power software (version 3.1.9.6, https://g-power.apponic.com) in this study. Data for the concentrations of immune and liver function indicators in serum were analyzed using repeated measures with week, treatment, block, and the interaction of treatment and week as the fixed effects, and the block and cow within diet as random effects. The effect of week was included as a repeated measure. The alpha diversity indices and the relative abundance of fecal microbial data were analyzed with treatment as a fixed effect, and block and cow within diet as random effects. Orthogonal polynomial contrasts were carried out to examine the linear and quadratic effects of different doses of calcium propionate by PROC MIXED of SAS (version 9.4, SAS Institute Inc., Cary, NC, USA). The adjusted contrast coefficients for unequal spacing of calcium propionate levels were generated by the IML procedure of SAS. Duncan's multiple range test was used for the evaluation of differences among the treatments. The statistically significant difference was considered at *P* ≤ 0.05, and the trend was declared at 0.05 < *P* ≤ 0.10.

## Results

### Serum immune and liver function indicators

The effects of calcium propionate supplementation on the serum parameters of immune and liver function of early-lactation dairy cows are shown in Table 2. Increasing doses of calcium propionate quadratically changed the concentrations of ALB (*P* = 0.03) and IgA (*P* = 0.01), while no difference occurred in serum TP, IgG, IgM, and Ca concentrations. The activities of serum ALT (*P* = 0.005) and AST (*P* < 0.001) showed linear increases with increasing calcium propionate supplementation, and all peaked in the HCaP group. While the concentration of serum ALP decreased linearly (*P* < 0.001) as the amount of calcium propionate increased.

**Table 2:** Effects of calcium propionate feeding levels on serum immune and liver function of dairy cows in early lactation.

**Items** ^1^	**Treatments** ^2^				**SEM** ^3^	***P*****-value** ^4^				
	**CON**	**LCaP**	**MCaP**	**HCaP**		**Trt**	**L**	**Q**	**Time**	**Trt** **×Time**
TP, g/L	70.51	69.55	72.46	70.78	0.57	0.24	0.45	0.89	<0.001	0.55
ALB, g/L	37.10 ^ab^	36.28 ^b^	37.02 ^ab^	38.66 ^a^	0.36	0.03	0.07	0.03	0.001	0.15
IgA, g/L	0.76	0.66	0.69	0.76	0.02	0.08	0.96	0.01	0.06	0.08
IgG, g/L	10.11	10.84	10.42	10.98	0.18	0.33	0.18	0.79	0.16	0.72
IgM, g/L	2.54	2.47	2.42	2.51	0.05	0.80	0.66	0.39	0.03	0.01
ALT, U/L	41.15 ^b^	41.51 ^b^	41.52 ^b^	45.61 ^a^	0.53	0.003	0.005	0.04	0.14	0.22
AST, U/L	58.81 ^c^	58.95 ^c^	62.57 ^b^	65.56 ^a^	0.84	<0.001	<0.001	0.02	<0.001	0.84
ALP, U/L	45.46 ^a^	43.58 ^ab^	43.2 ^b^	40.05 ^c^	0.67	<0.001	<0.001	0.26	<0.001	<0.001
Ca, mmol/L	2.23	2.33	2.29	2.39	0.04	0.68	0.30	0.99	0.50	0.58

^a, b, c^ Means within a row with different superscripts differ significantly (P ≤ 0.05).

^1^TP, total protein; ALB, albumin; IgA, immunoglobulin A; IgG, immunoglobulin G; IgM, immunoglobulin M; ALT, alanine transaminase; AST, aspartate aminotransferase; ALP, alkaline phosphatase; Ca, calcium.

^2^CON, control group; LCaP, low calcium propionate group, basal diet + 200 g/d calcium propionate; MCaP, medium calcium propionate group, basal diet + 350 g/d calcium propionate; HCaP, high calcium propionate group, basal diet + 500 g/d calcium propionate.

^3^SEM, Standard error of the mean.

^4^ Trt, Contrast Among CON, LCaP, MCaP, and HCaP; L, Linear Effect of Calcium Propionate Addition; Q, Quadratic Effect of Calcium Propionate Addition; Time, Time Effect; Trt × Time, the Interaction Effect Between Treatment and Time.

### Fecal bacteria richness, diversity, and community composition

In total, 2,376,621 raw reads were obtained in the fecal samples of the four groups. After data filtering, quality checking, and chimera removal, 2,310,615 high-quality 16S rRNA gene sequences were obtained from the 24 fecal samples, averaging 96,276 sequences reads for each sample (ranging from 35,377 to 171,181 sequences), which were used for the downstream analyses. The lengths of the sequence reads were mainly distributed in 400-440 bp (Supplementary figure S1). The sample-based rarefaction curves showed that with the number of reads per sample increased (Supplementary figure S2), the identified new OUTs showed a diminishing rate, implying that the sequencing depth had sufficient coverage to accurately describe the fecal bacterial community composition. After sub-sampling and clustering, a total of 2,402 OTUs were obtained in the four groups based on ≥97% nucleotide sequence identity between reads. The OUT numbers of CON, LCaP, MCaP, and HCaP were 1,995, 2,039, 1,985, and 1,971, respectively. The Venn diagram showed that 1,573 OTUs were shared across the four groups (Supplementary figure S3), representing 65.4% of total OUTs detected across all samples. The Good's coverage values for all samples averaged 99%, which indicated the high accuracy and reproducibility of the sequencing.

**Figure 1:**
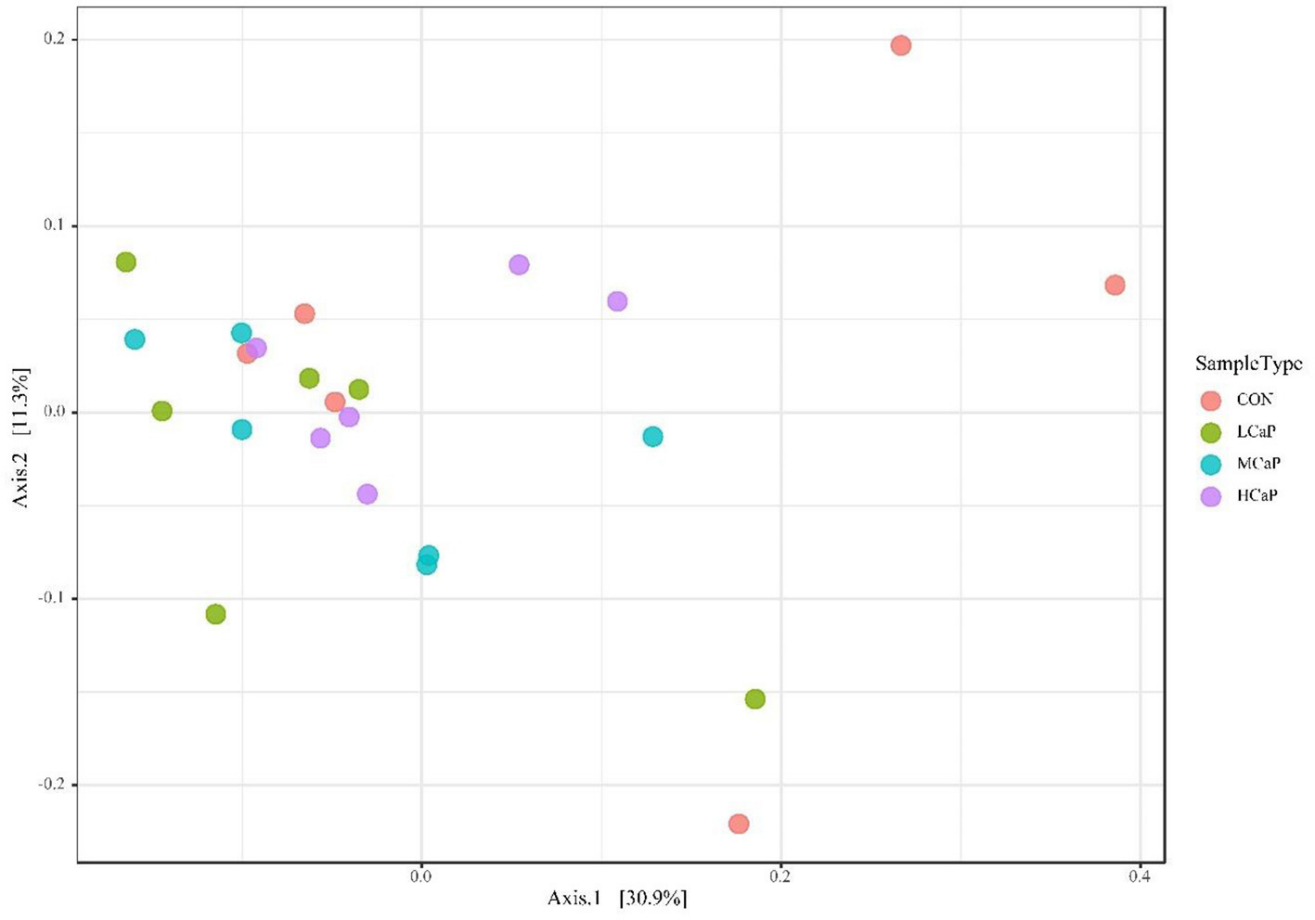
Principal coordinates analysis (PCoA) of beta diversity using Bray-Curtis distances in the fecal bacterial communities. Each point of the PCoA represents a sample among the different levels of calcium propionate treatments. The greater similarity in the bacterial communities of the samples, the lower distance of the points. CON, control group; LCaP, low calcium propionate group, basal diet + 200 g/d calcium propionate; MCaP, medium calcium propionate group, basal diet + 350 g/d calcium propionate; HCaP, high calcium propionate group, basal diet + 500 g/d calcium propionate.

**Figure 2:**
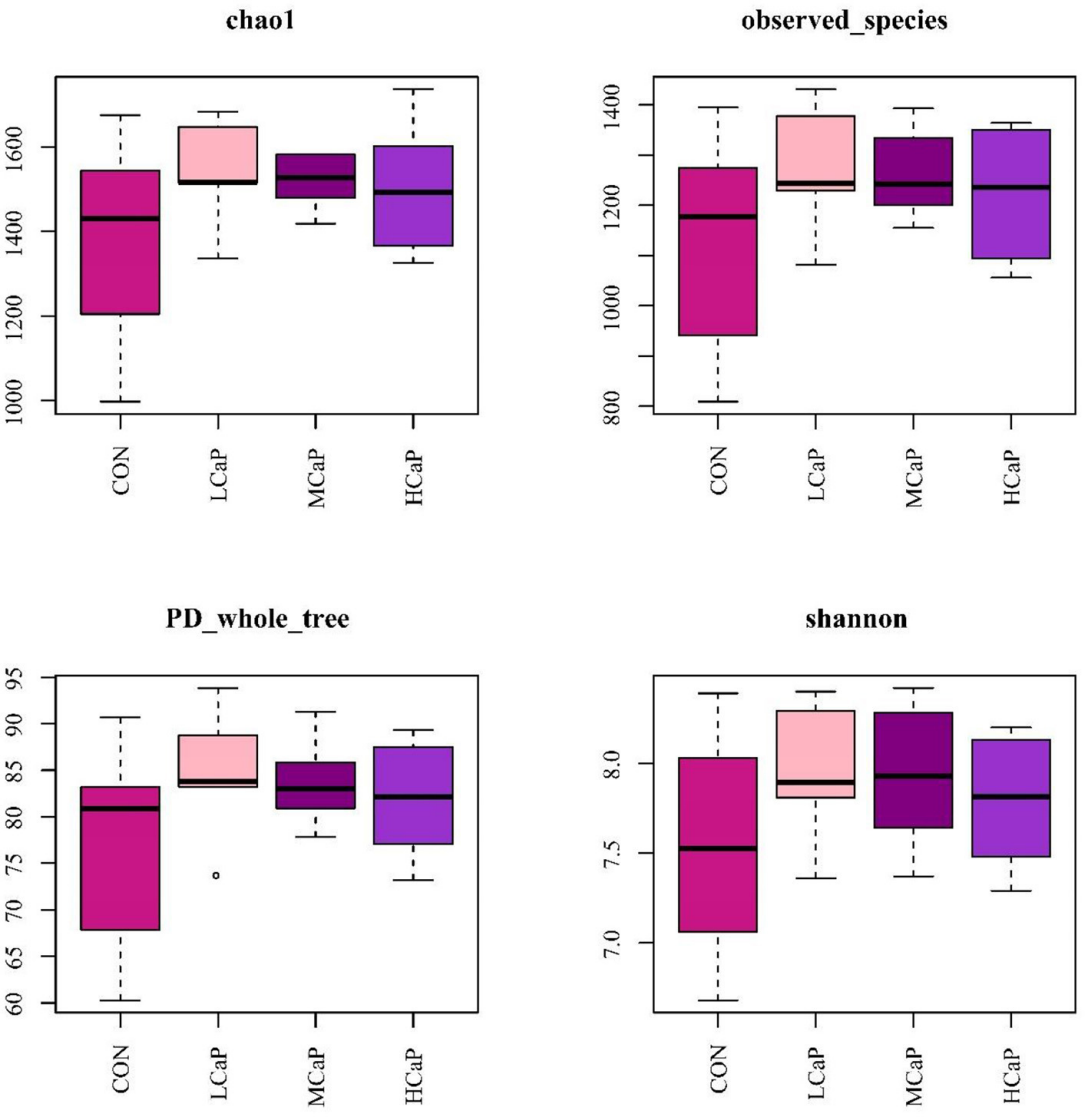
Box-and-whisker plots of alpha diversity indices in the fecal bacterial communities in different levels of calcium propionate treatments. CON, control group; LCaP, low calcium propionate group, basal diet + 200 g/d calcium propionate; MCaP, medium calcium propionate group, basal diet + 350 g/d calcium propionate; HCaP, high calcium propionate group, basal diet + 500 g/d calcium propionate.

The β-diversity analysis was used to explore the differences in the fecal bacterial community among the 4 groups. PCoA plots based on Bray Curtis distance matrices showed no apparent clustering of bacterial community among the treatments (Figure 1). Furthermore, the alpha diversity indices are presented in Figure 2 and Supplementary table S1. The results of Chao 1, observed species, PD_whole_tree, Shannon, and Simpson indices were not affected by the dietary supplementation of calcium propionate to the dairy cows in early lactation.

**Figure 3:**
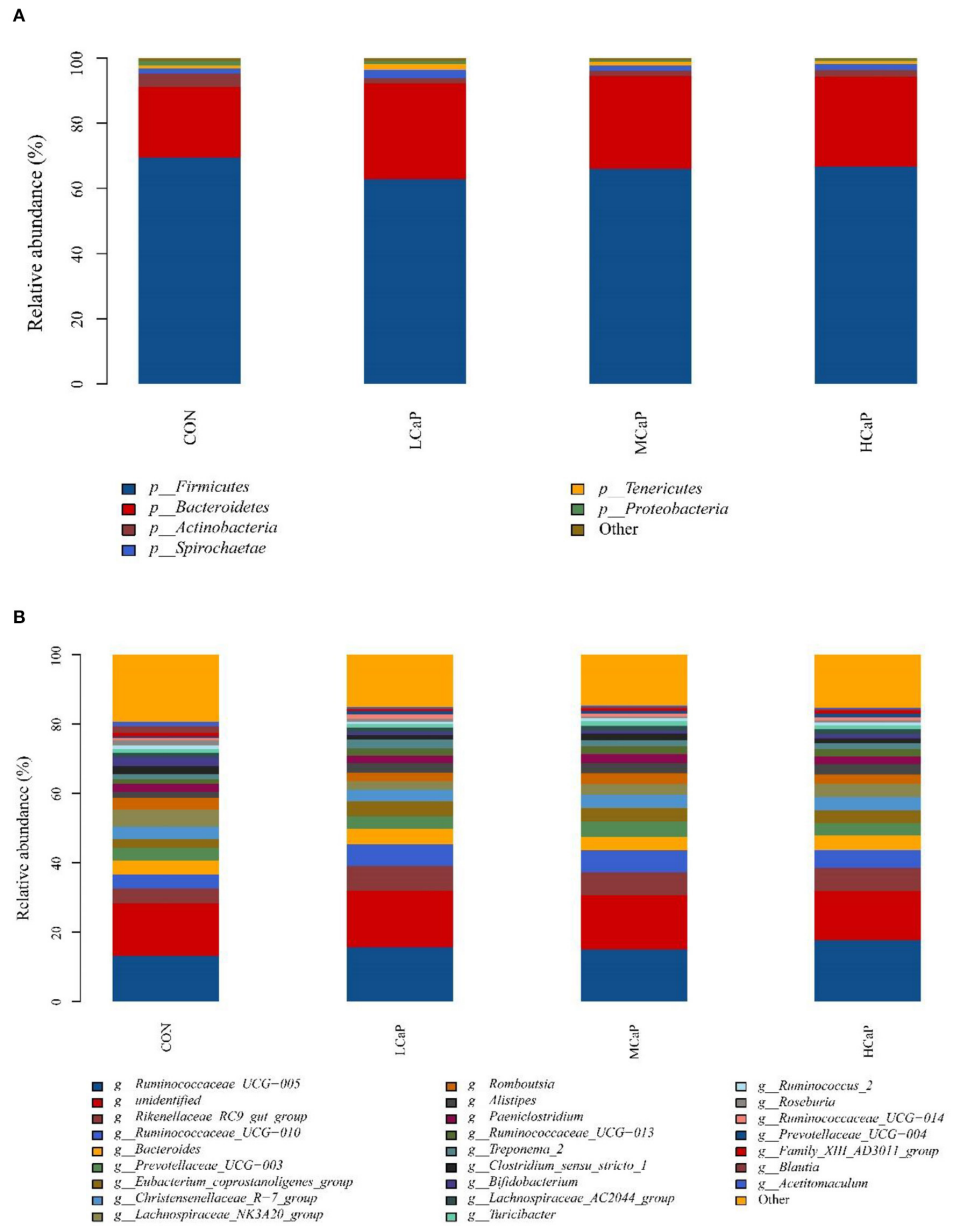
Distribution of the fecal bacterial community composition under phyla **(A)** and genera **(B)** levels across the different levels of calcium propionate treatments. CON, control group; LCaP, low calcium propionate group, basal diet + 200 g/d calcium propionate; MCaP, medium calcium propionate group, basal diet + 350 g/d calcium propionate; HCaP, high calcium propionate group, basal diet + 500 g/d calcium propionate.

The SILVA taxonomic database was used to classify all the sequences from phylum to species. A total of 17 phyla of bacteria were detected in the fecal samples. In which, *Firmicutes* and *Bacteroidetes* were the predominant phyla, representing 66.26 ± 1.03% (mean ± SEM) and 26.87 ± 1.11% of the total sequences, respectively (Figure 3A). Other bacterial phyla including *Actinobacteria, Spirochaetae, Tenericutes*, and *Proteobacteria*, had relative abundances averaged at 2.29 ± 0.41%, 1.89 ± 0.23%, 1.24 ± 0.14%, and 0.78 ± 0.26%, respectively. The rest phyla represented <0.5 % of all sequences. At the genus level, a total of 238 bacterial genera were identified in the 24 dairy cows' fecal samples, and 83 genera appeared across all samples. The relative abundances of the 10 predominant genera were *Ruminococcaceae_UCG-005* (15.40 ± 0.63%), *Rikenellaceae_RC9_gut_group* (6.19 ± 0.39%), *Ruminococcaceae_UCG-010* (5.43 ± 0.52%), *Bacteroides* (4.15 ± 0.27%), *Prevotellaceae_UCG-003* (3.83 ± 0.38%), *Eubacterium_coprostanoligenes_group* (3.62 ± 0.34%), *Christensenellaceae_R-7_group* (3.61 ± 0.17%), *Lachnospiraceae_NK3A20_group* (3.57 ± 0.27%), *Romboutsia* (2.92 ± 0.28%), and *Alistipes* (2.59 ± 0.21%) (Figure 3B).

### Differences in fecal bacterial community composition

At the phylum level (Table 3), increasing levels of calcium propionate tended to quadratically decrease the relative abundance of *Firmicutes* (*P* = 0.06) and *Actinobacteria* (*P* = 0.07). Conversely, the relative abundance of *Bacteroidetes* was quadratically (*P* = 0.04) increased with the increasing calcium propionate addition. Moreover, the relative abundances of *Bacteroidetes* in the calcium propionate treatment groups were all significantly higher than that in the CON group (*P* = 0.04). No treatment effect was observed on the abundance of phyla *Spirochaetae, Tenericutes*, and *Proteobacteria*. Increasing levels of calcium propionate linearly decreased the ratio of *Firmicutes* to *Bacteroidetes* (*P* = 0.04).

**Table 3:** Effects of calcium propionate feeding levels on the relative abundances (%) of major fecal bacterial communities at the phylum level (averagely accounting for ≥ 0.5% of the total sequences in at least one treatment) of dairy cows in early lactation.

**Phylum**	**Treatments** ^1^				**SEM** ^2^	***P*****-value** ^3^		
	**CON**	**LCaP**	**MCaP**	**HCaP**		**Trt**	**L**	**Q**
*Firmicutes*	69.53	62.81	66.02	66.69	1.03	0.14	0.44	0.06
*Bacteroidetes*	21.75 ^b^	29.56 ^a^	28.52 ^a^	27.66 ^a^	1.11	0.04	0.05	0.04
*Actinobacteria*	3.97	1.45	1.61	2.13	0.41	0.10	0.10	0.07
*Spirochaetae*	1.53	2.72	1.59	1.73	0.23	0.21	0.89	0.18
*Tenericutes*	1.06	1.65	1.25	1.00	0.14	0.37	0.74	0.12
*Proteobacteria*	1.37	1.00	0.41	0.32	0.26	0.45	0.12	0.90
*Firmicutes*/*Bacteroidetes*	3.90	2.16	2.32	2.44	0.27	0.06	0.04	0.08

^a, b^ Means within a row with different superscripts differ significantly (P ≤ 0.05).

^1^CON, control group; LCaP, low calcium propionate group, basal diet + 200 g/d calcium propionate; MCaP, medium calcium propionate group, basal diet + 350 g/d calcium propionate; HCaP, high calcium propionate group, basal diet + 500 g/d calcium propionate.

^2^SEM, Standard error of the mean.

^3^Trt, contrast among CON, LCaP, MCaP, and HCaP; L, linear effect of calcium propionate addition; Q, quadratic effect of calcium propionate addition.

The relative abundances of the 24 most abundant bacterial genera (averagely accounting for ≥ 0.5% of the total sequences in at least one treatment) are shown in Table 4, which belonged to *Firmicutes, Bacteroidetes, Spirochaetae*, and *Actinobacteria*. The proportion of *Ruminococcaceae_UCG-005*, the most predominant genus, was linearly (*P* = 0.02) increased with increasing supplementation of calcium propionate. The relative abundances of *Lachnospiraceae_NK3A20_group* (*P* < 0.001), and *Family_XIII_AD3011_group* (*P* = 0.01) were quadratically changed with the increasing levels of calcium propionate, and the lowest values were both observed in the LCaP group. However, the relative abundance of *Ruminococcaceae_UCG-014* in LCaP was significantly higher than the other groups (*P* = 0.03). Compared with the CON group, the calcium propionate supplementation significantly decreased the relative abundance of *Acetitomaculum* (*P* = 0.02) and increased the abundances of *Rikenellaceae_RC9_gut_group* (*P* = 0.05) and *Alistipes* (*P* = 0.05). Meanwhile, increasing doses of calcium propionate linearly increased the relative abundance of *Prevotellaceae_UCG-004* (*P* = 0.05). The other genera with relative abundances above 0.5% showed no significant difference among the four treatments.

**Table 4:** Effects of calcium propionate feeding levels on relative abundance (%) of major fecal bacterial communities at the genus level (averagely accounting for ≥ 0.5% of the total sequences in at least one treatment) of dairy cows in early lactation.

**Phylum**	**Genus**	**Treatments** ^1^				**SEM** ^2^	***P*****-value** ^3^		
		**CON**	**LCaP**	**MCaP**	**HCaP**		**Trt**	**L**	**Q**
*Firmicutes*	*Ruminococcaceae_UCG-005*	13.17	15.69	15.05	17.71	0.63	0.07	0.02	0.90
	*Ruminococcaceae_UCG-010*	3.95	6.20	6.32	5.24	0.52	0.37	0.34	0.14
	*Eubacterium_coprostanoligenes_group*	2.60	4.27	3.91	3.69	0.34	0.35	0.29	0.18
	*Lachnospiraceae_NK3A20_group*	4.95 ^a^	2.40 ^c^	3.14 ^bc^	3.79 ^ab^	0.27	0.002	0.09	<0.001
	*Christensenellaceae_R-7_group*	3.58	3.35	3.67	3.85	0.17	0.80	0.52	0.52
	*Ruminococcaceae_UCG-013*	1.31	1.96	2.30	2.11	0.19	0.29	0.09	0.33
	*Blautia*	1.88	0.23	0.28	0.30	0.35	0.29	0.12	0.27
	*Romboutsia*	3.42	2.42	3.16	2.67	0.28	0.59	0.51	0.61
	*Paeniclostridium*	2.28	2.17	2.67	2.25	0.24	0.90	0.86	0.83
	*Ruminococcaceae_UCG-014*	0.65 ^b^	1.25 ^a^	0.74 ^b^	0.72 ^b^	0.08	0.03	0.79	0.03
	*Ruminococcus_2*	1.01	0.81	0.89	0.90	0.07	0.84	0.68	0.49
	*Family_XIII_AD3011_group*	1.03 ^a^	0.58 ^b^	0.82 ^ab^	0.90 ^a^	0.06	0.04	0.61	0.01
	*Ruminococcaceae_NK4A214_group*	0.64	0.67	0.69	0.74	0.03	0.71	0.26	0.84
	*Clostridium_sensu_stricto_1*	2.31	1.22	1.97	1.37	0.27	0.46	0.36	0.62
	*Acetitomaculum*	1.36 ^a^	0.28 ^b^	0.43 ^b^	0.41 ^b^	0.15	0.02	0.02	0.06
	*Lachnospiraceae_AC2044_group*	1.20	1.29	1.38	1.47	0.18	0.96	0.61	0.97
	*Turicibacter*	1.20	0.89	1.33	1.04	0.14	0.75	0.95	0.90
*Bacteroidetes*	*Rikenellaceae_RC9_gut_group*	4.41 ^b^	7.21 ^a^	6.49 ^a^	6.64 ^a^	0.39	0.05	0.05	0.08
	*Prevotellaceae_UCG-003*	3.53	3.74	4.52	3.52	0.38	0.79	0.81	0.50
	*Bacteroides*	4.05	4.47	3.90	4.16	0.27	0.91	0.95	0.84
	*Alistipes*	1.63 ^b^	2.85 ^a^	2.87 ^a^	2.99 ^a^	0.21	0.05	0.02	0.19
	*Prevotellaceae_UCG-004*	0.46	0.91	0.80	1.16	0.11	0.19	0.05	0.87
*Spirochaetae*	*Treponema_2*	1.51	2.68	1.55	1.70	0.23	0.22	0.87	0.19
*Actinobacteria*	*Bifidobacterium*	2.61	0.90	0.86	1.20	0.36	0.27	0.15	0.17

^a, b, c^ Means within a row with different superscript letters differ significantly (P ≤ 0.05).

^1^CON, control group; LCaP, low calcium propionate group, basal diet + 200 g/d calcium propionate; MCaP, medium calcium propionate group, basal diet + 350 g/d calcium propionate; HCaP, high calcium propionate group, basal diet + 500 g/d calcium propionate.

^2^SEM, Standard error of the mean.

^3^Trt, contrast among CON, LCaP, MCaP, and HCaP; L, linear effect of calcium propionate addition; Q, quadratic effect of calcium propionate addition.

**Figure 4:**
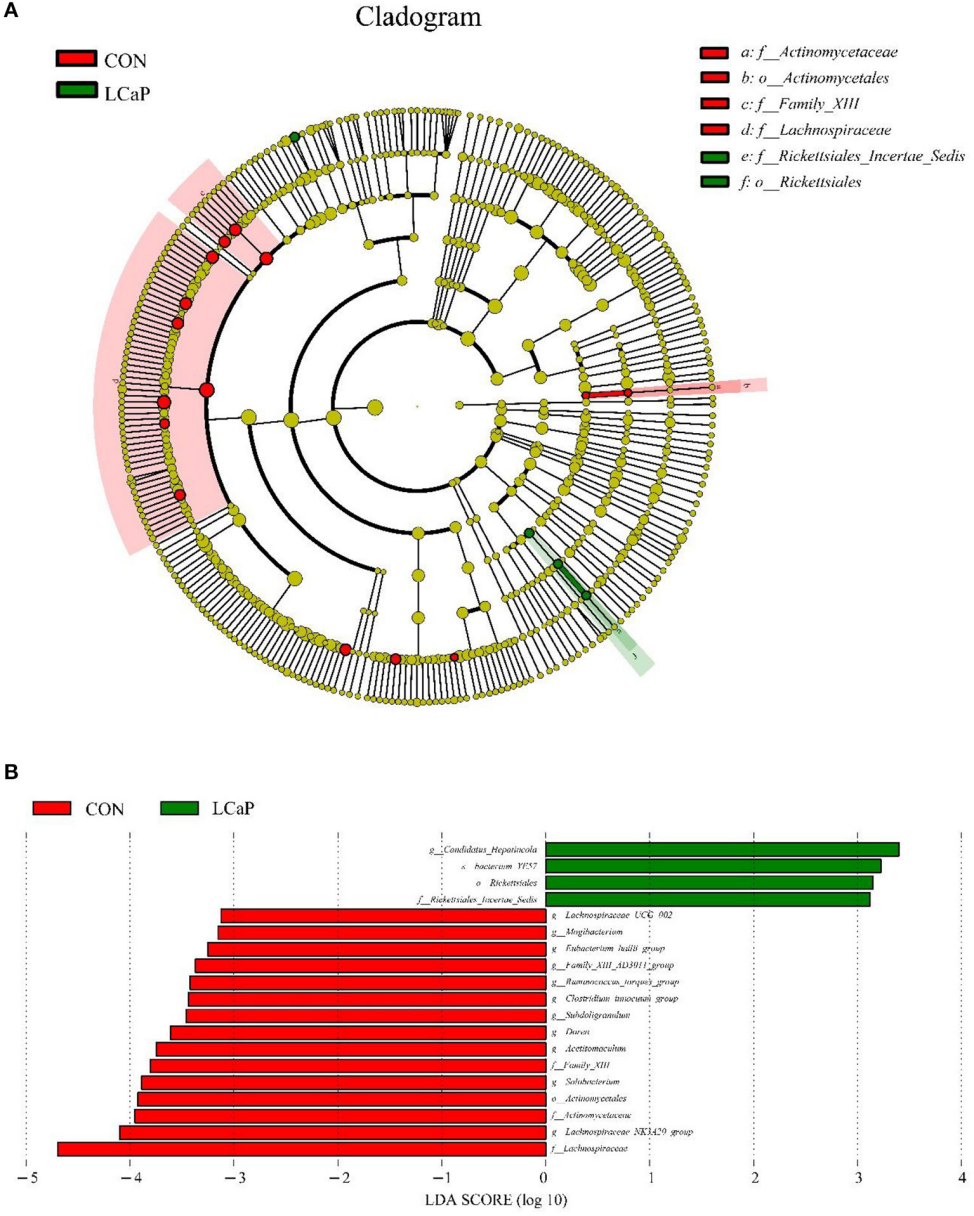
Linear discriminant analysis effect size (LEfSe) analysis of significant differences in fecal bacterial community composition between the CON and LCaP groups. **(A)** LEfSe analysis-derived taxonomic cladogram. **(B)** Linear discriminant analysis (LDA) scores. Significant differences were defined as LDA scores > 3 and *P* < 0.05. CON, control group; LCaP, low calcium propionate group, basal diet + 200 g/d calcium propionate.

The results showed that, compared with the CON group, LCaP treatment significantly changed the fecal microbiota. The linear discriminant analysis effect size (LEfSe) analysis revealed 19 differentially abundant taxonomic clades (linear discriminant analysis (LDA) score > 3), with 15 in the CON group, and 4 in the LCaP group (Figure 4). The family *Lachnospiraceae*, genus *Lachnospiraceae_NK3A20_group*, family *Actinomycetaceae*, and order *Actinomycetales* were the key communities in the CON group, while the genus *Candidatus_Hepatincola*, species *bacterium_YE57*, order *Rickettsiales*, and family *Rickettsiales_Incertae_Sedis* were enriched in the LCaP group (Figure 4). There was no species substantially enriched in the MCaP or HCaP groups.

## Discussion

Dietary supplementation with calcium propionate is one of the effective strategies to prevent ketosis and milk fever metabolic disorders in early lactation dairy cows. Our previous study showed that the addition of calcium propionate could quadratically improve the milk performance of dairy cows in early lactation, peaking at the 350 g/d feeding level (19). In the current study, we aimed to elucidate the changes of immune function and fecal bacteria community of early lactation dairy cows supplemented with different levels of calcium propionate.

The concentrations of serum proteins, including ALB (35–50% of total protein) and immune globulins (IgA, IgG, and IgM), are widely used as diagnostic tools to assess the inflammatory status and predict the outcome of severe diseases in humans and animals (29). In the present study, the serum TP, IgG, and IgM concentrations were not affected by the supplementation of calcium propionate, but the concentrations of ALB and IgA were quadratically changed with the increasing feeding levels of calcium propionate. The production of ROS may impair immune cell function and compromise the defenses of the immune system against invading bacteria (30). The results of Jiao et al. (31) also suggested that immunity was positively correlated with antioxidant capacity. In the present study, the ALB concentrations in the calcium propionate treatment groups were not significantly different with the CON group. However, its concentration in the LCaP group was lower than that in the HCaP group. Therefore, we speculated that the quadratically changed serum IgA and ALB concentrations may be related to the quadratically altered milk yield and antioxidant capacity (19).

The concentrations of AST, ALT, and ALP in the blood are sensitive markers of liver damage (32). The results of Wang et al. (33) showed that supplementation of rumen-protected glucose to the dairy cows in early lactation at 350 g/d could significantly decrease the serum AST, but the milk yield did not increase. Li et al. (34) discovered that supplementation with 200 g/d rumen-protected glucose could improve milk production, but tended to increase plasma ALT. In the present study, the increasing serum AST in MCaP may be related to the higher milk yield in this group. The serum ALT and AST levels in the HCaP group were both significantly higher than those in the other groups, indicating decreased hepatic function. Moreover, the ALP level was linearly decreased with the increasing levels of calcium propionate. The level of ALP is not only related to liver function but also connected with calcium metabolism. Yoshimura et al. (35) discovered that a low-calcium environment could stimulate the ALP activity of bone cells to maintain normal cell function. Therefore, the lower serum ALP in the treatment groups could be resulted from the increasing supplementation of calcium and consequently decreased the bone calcium turnover. The oral treatment with calcium propionate contributed to improving Ca absorption from diet by passive diffusion (36). While in this study, the dietary supplementation with calcium propionate didn't significantly improve the serum Ca concentration, which may be associated with the higher bone calcium mobilization in the CON group. The results of Goff et al. (15) showed that calcium propionate treatment had no significant effects on blood Ca of dairy cows but reduced the incidence of milk fever, which were similar to our results. Ramella et al. (37) also discovered that oral treatment with calcium formate did not improve the serum Ca concentration of early lactation dairy cows.

Feeding calcium propionate affected the fecal bacterial community of dairy cows in the current study. It is well known that the structure of fecal microbiome is significantly influenced by diet (38, 39) and plays a critical role in the host animal health through the immune system. Therefore, investigating the changes in fecal microbiota community and composition contribute to understanding the effects of calcium propionate on the health status of dairy cows. In the current study, although the fecal bacterial diversity indices were not significantly different, the calcium propionate treated groups had a numerically higher value than the CON group. Therefore, the supplementation of calcium propionate did not lead to the dysbiosis of fecal bacterial diversity.

Similar with the previous studies (40, 41), the most abundant fecal bacteria communities at the phylum level were *Firmicutes* and *Bacteroidetes*, together representing more than 90% of the total bacterial population. In the present study, we found that diet supplementation with increasing amounts of calcium propionate tended to quadratically decrease the relative abundance of *Firmicutes*, while quadratically increased the abundance of *Bacteroidetes*, and consequently linearly decreased the *Firmicutes*/*Bacteroidetes* ratio. According to the report of Fan et al. (42), exogenous supplementation of propionate significantly restored the abundance of *Bacteroidetes* in the feces of arthritic rats, which was in agreement with the current results. The percentage of *Bacteroidetes* is positively correlated with the plasma glucose concentration (43). The results of Chen et al. (44) suggested that the specific species of *Firmicutes* may induce more hepatic steatosis by modulating fatty acid influx and lipogenesis compared to those of *Bacteroidetes*. The incidence of fatty liver is particularly high in the high-yielding dairy cows during the postpartum period for the excessive triglycerides deposit in the liver (45). The shifted relative abundances of *Firmicutes* and *Bacteroidetes* may explain the effect of calcium propionate in alleviating NEB. Colonization of *Firmicutes* could contribute to the activation of osteoclasts and the exacerbation of inflammation (46). As a member of polysaccharide-degrading consortia, the increase abundance of *Bacteroidetes* contributed to the release of energy from dietary fiber and starch, which might also help to suppress inflammation (43) and decrease the pro-inflammatory cytokines production (47). The decrease of *Firmicutes*/*Bacteroidetes* ratio in the calcium propionate treatment groups suggested the decrease in gut inflammation response and bone degradation, which could therefore prevent the occurrence of osteoporosis. Moreover, the abundance of the gut bacterial phylum *Bacteroidetes* was responsible for the production of secondary bile acids (48). Therefore, the increased serum bile acids constituents in the calcium propionate treatment groups in our previous study (19) may be related to the increased percentage of *Bacteroidetes* in the gut.

At the genus level, similar to the study of Castillo-Lopez et al. (49), we found that *Ruminococcaceae_UCG-005, Ruminococcaceae_UCG-010*, and *Rikenellaceae_RC9_gut_group* were the most abundant genera in the fecal bacteria community of dairy cows. The relative abundance of the family *Ruminococcaceae*, such as the genus *Ruminococcaceae_UCG-005, Ruminococcaceae_UCG-010, Ruminococcaceae_UCG-013*, and *Ruminococcaceae_UCG-014*, were all higher in the calcium propionate treatment groups than the CON group. The abundances of family *Ruminococcaceae* were 28.34, 35.73, 34.92, and 35.95% for CON, LCaP, MCaP, and HCaPgroups, respectively, (linearly increased with *P* = 0.03, data not shown). The members of *Ruminococcaceae* contribute to the metabolism of fiber. Therefore, the increased relative abundance of *Ruminococcaceae* in the calcium propionate supplementation groups indicated the improvement of fiber digestion in the gut (50). In the current study, we found that dietary supplementation with calcium propionate quadratically decreased the abundance of *Lachnospiraceae_NK3A20_group*, which may decrease the production of butyrate in the gut (51). Besides, the genera of *Rikenellaceae_RC9_gut_group* and *Alistipes* were increased significantly in the calcium propionate treatment groups. The study of Ma et al. (52) showed that the relative abundance of *Rikenellaceae_RC9_gut_group* in the ileum of the normal yaks was higher than that of the growth-retarded yaks. The abundance of cecal *Rikenellaceae_RC9_gut_group* was negatively correlated with the diarrhea rate (53, 54). The genus of *Alistipes* has protective effects against many diseases and promote the phenotypes of health (55). Therefore, the increased abundances of *Rikenellaceae_RC9_gut_group* and *Alistipes* in the calcium propionate treatment groups could contribute to the health of the dairy cows. Detecting the changes of the hindgut bacterial population provided a useful reference point to understand the potential function of dietary calcium propionate supplementation. However, the potential mechanisms for the association between the serum parameters and fecal bacterial taxa abundances still need further investigation.

## Conclusions

Overall, the present study demonstrated that dietary calcium propionate supplementation quadratically changed serum ALB and IgA concentrations. The liver function was decreased for the dairy cows supplemented with 500 g/d calcium propionate during the first 35 d of lactation. Analysis of fecal bacteria community structure revealed that calcium propionate supplementation significantly improved the relative abundances of *Bacteroidetes*, including *Rikenellaceae_RC9_gut_group* and *Alistipes*. Calcium propionate supplementation to the dairy cows in early lactation contributed to decreasing gut inflammation response and could possibly improve gut nutrient digestion. This study provided novel evidences for the effects of calcium propionate on the health and intestinal bacterial structure of dairy cows in the process of alleviating negative nutrition balance in early lactation.

## Data availability statement

The datasets presented in this study can be found in online repositories. The names of the repository/repositories and accession number(s) can be found in the article/Supplementary material.

## Ethics statement

The animal study was reviewed and approved by Animal Care Committee of the Chinese Academy of Agricultural Sciences (Beijing, China).

## Author contributions

FZ: conceptualization, investigation, data curation, and writing–original draft. YZ, HW, and XN: writing–review and editing. YW: investigation and methodology. YG: conceptualization and supervision. BX: project administration, funding acquisition, conceptualization, and supervision. All authors read and approved the final manuscript.

## Funding

This study was financially supported by the National Key R&D Program of China (2019YFE0125600 and 2021YFD2000804), Science and Technology Innovation 2030-Key Project of China (2021ZD0113801), and State Key Laboratory of Animal Nutrition (2004DA125184G2204).

## Conflict of interest

The authors declare that the research was conducted in the absence of any commercial or financial relationships that could be construed as a potential conflict of interest.

## Publisher's note

All claims expressed in this article are solely those of the authors and do not necessarily represent those of their affiliated organizations, or those of the publisher, the editors and the reviewers. Any product that may be evaluated in this article, or claim that may be made by its manufacturer, is not guaranteed or endorsed by the publisher.
