# Supplementary material for: Dietary supplementation with calcium propionate could beneficially alter rectal microbial composition of early lactation dairy cows
Source: Front Vet Sci. 2022 Jul 26;9:940216. doi: 10.3389/fvets.2022.940216 (PMC9360568; doi:10.3389/fvets.2022.940216)
Supplement: Supplementary file 1 [file Data_Sheet_1.docx]

Dietary Supplementation with Calcium Propionate Could Beneficially Alter Rectal Microbial Composition of Early Lactation Dairy Cows

Fan Zhang^1,2,†^, Yiguang Zhao ^1,†^, Yue Wang^1^, Hui wang^1^, Xuemei Nan^1^, Yuming Guo^2^, and Benhai Xiong^1,*^

^1^State Key Laboratory of Animal Nutrition, Institute of Animal Sciences, Chinese Academy of Agricultural Sciences, Beijing 100193, China

^2^State Key Laboratory of Animal Nutrition, College of Animal Science and Technology, China Agricultural University, Beijing 100193, China

*****Corresponding author: Yuming Guo, Email: guoyum@cau.edu.cn; Benhai Xiong, Email: [xiongbenhai@caas.cn](mailto:xiongbenhai@caas.cn).

^†^These authors contributed equally to this work.

**
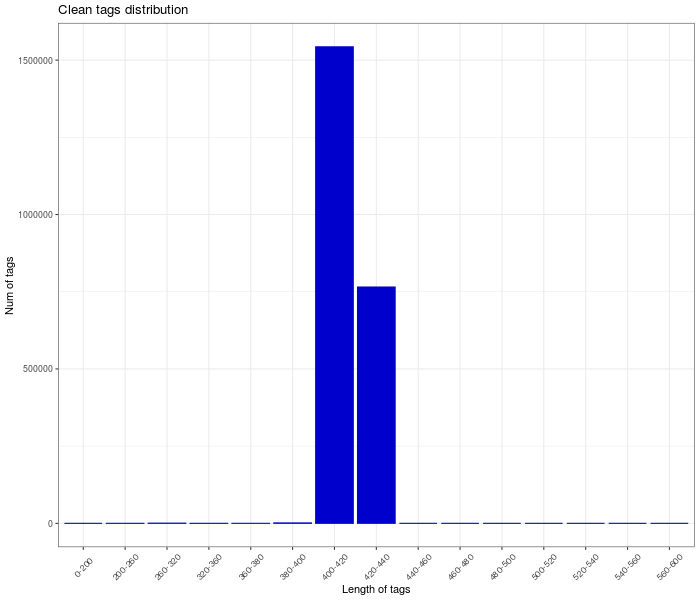
Supplementary Figure S1** Distribution statistics of high-quality 16S rRNA gene sequences of bacteria in rectal fecal samples.


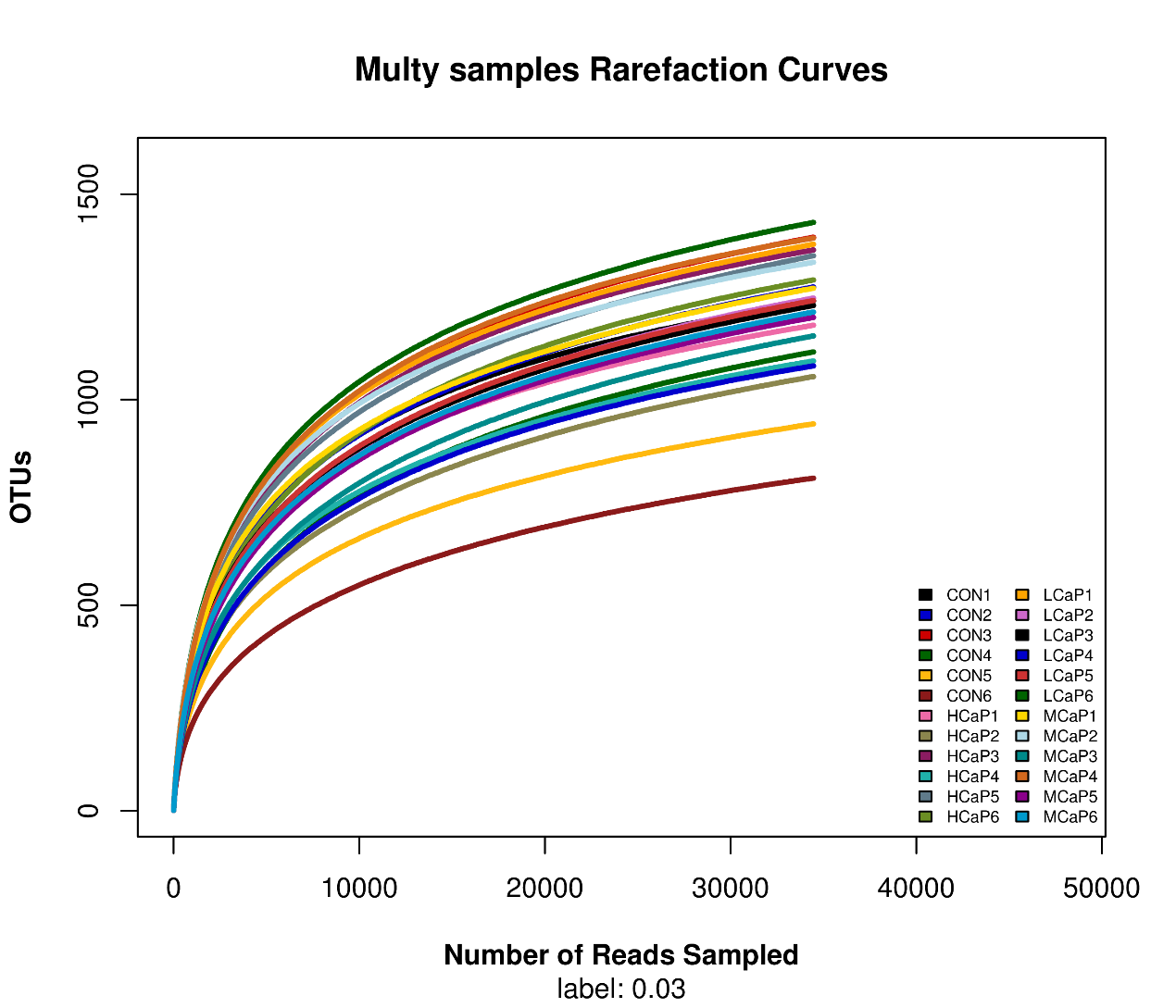
**Supplementary Figure S2** Rarefaction curves for the relationships between Operational taxonomic units (sharing ⩾ 97% sequence identity) and sequences of each rectal fecal sample across different levels of calcium propionate treatments. CON: control group; LCaP: low calcium propionate group, basal diet + 200 g/d calcium propionate; MCaP: medium calcium propionate group, basal diet + 350 g/d calcium propionate; HCaP: high calcium propionate group, basal diet + 500 g/d calcium propionate.


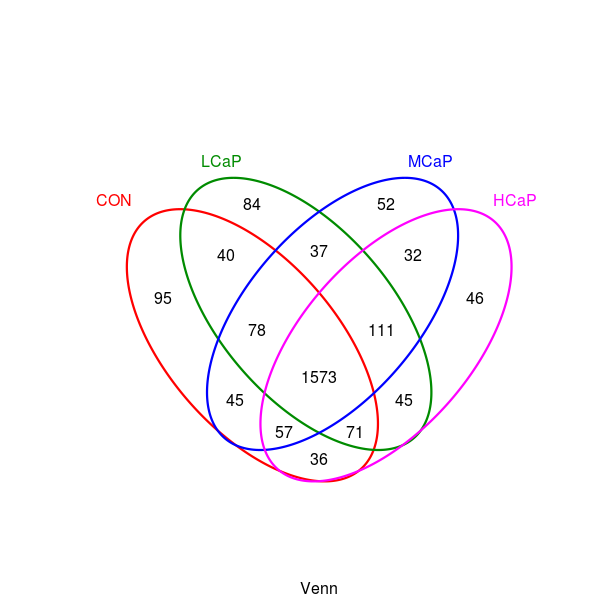


**Supplementary Figure S3** Venn diagram of operational taxonomic unit (OTU) relationships between treatments. Each cycle represents a type of treatment. Numbers in overlapping areas represent the shared OTU across treatment groups. CON: control group; LCaP: low calcium propionate group, basal diet + 200 g/d calcium propionate; MCaP: medium calcium propionate group, basal diet + 350 g/d calcium propionate; HCaP: high calcium propionate group, basal diet + 500 g/d calcium propionate.

**Supplementary Table S1**. Effects of calcium propionate feeding levels on the alpha diversity indices of fecal bacterial communities in early lactation dairy cows.

| Items | Treatment ^1^ | | | | SEM ^2^ | *P*-value ^3^ | | |
| --- | --- | --- | --- | --- | --- | --- | --- | --- |
|  | CON | LCaP | MCaP | HCaP |  | Trt | L | Q |
| Chao 1 | 1 380 | 1 536 | 1 520 | 1 503 | 33.26 | 0.35 | 0.21 | 0.22 |
| Observed_species | 1 129 | 1 268 | 1 261 | 1 223 | 30.65 | 0.37 | 0.28 | 0.17 |
| PD_whole_tree | 77.29 | 84.54 | 83.63 | 81.89 | 1.58 | 0.39 | 0.32 | 0.18 |
| Shannon | 7.54 | 7.94 | 7.93 | 7.79 | 0.09 | 0.42 | 0.34 | 0.18 |
| Simpson | 0.98 | 0.99 | 0.98 | 0.98 | 0.001 | 0.57 | 0.99 | 0.17 |

^1^ Treatments: CON: control group; LCaP: low calcium propionate group, basal diet + 200 g/d calcium propionate; MCaP: medium calcium propionate group, basal diet + 350 g/d calcium propionate; HCaP: high calcium propionate group, basal diet + 500 g/d calcium propionate.

^2^ SEM = Standard error of the mean.

^3^ Trt = contrast between CON, LCaP, MCaP, and HCaP; L = linear effect of calcium propionate addition, Q = quadratic effect of calcium propionate addition.
